# Genetics in Macedonia—Following the international trends

**DOI:** 10.1002/mgg3.372

**Published:** 2018-02-20

**Authors:** Elena Sukarova – Angelovska, Aleksandar Petlichkovski

**Affiliations:** ^1^ University Clinic for Children's Diseases Medical Faculty, University Sv. Kiril i Metodij Skopje Republic of Macedonia; ^2^ Institute for Immunobiology and Human Genetics Medical Faculty, University Sv. Kiril i Metodij Skopje Republic of Macedonia

## Abstract

Genetics in Macedonia—Following the international trends

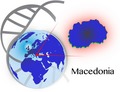

## 1 MACEDONIA—A CROSSROADS OF THE BALKANS

**Figure 1:**
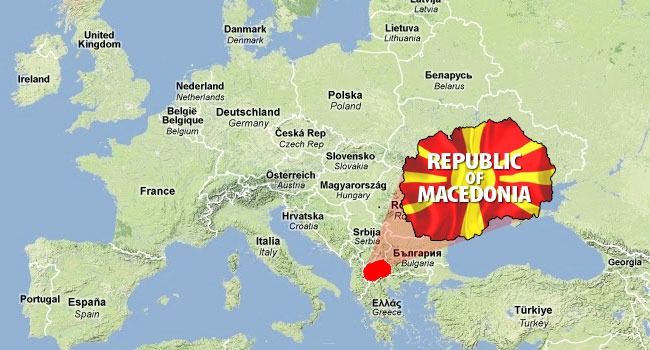
Geolocation of Macedonia in the heart of Balkan Peninsula

Macedonia is a small European country situated in the central part of the Balkan Peninsula and it borders other countries in the peninsula—Serbia, Bulgaria, Greece, Albania, and Kosovo (Figure 1). It covers a surface area of about 25.713 km^2^ and the landscape is predominantly hilly and mountainous. Macedonia gained its independence as one of the six Yugoslav republics in 1992, and today, most of its citizens perceive the aspiration for further development in the European integration processes.

Under the latest estimation of December 2015, the total population of the country is 2 071 278 inhabitants (Statistics, 2015). The population is multi‐ethnic and according to the last census in 2002 (Statistics, 2002), most of the population (about 64%) declared themselves as Macedonians, while the other ethnic groups included Albanians, Turks, Roma, Serbs, Bosniaks, and Vlachs (Table 1). Most of the inhabitants (Macedonians, Serbs, Vlachs) are Orthodox Christians and the second major group (Albanians, Turks, Bosniaks) belongs to the Muslim religion.

**Table 1:** Population in Macedonia according to ethnic distribution

Total (%)	Macedonians (%)	Albanians (%)	Turks (%)	Roma (%)	Serbs (%)	Bosniaks (%)	Vlachs (%)	Other (%)
2022547 (100)	1297981 (64.2)	509083 (25.2)	77959 (3.9)	53879 (2.8)	35939 (1.8)	17018 (0.8)	9695 (0.5)	20993 (1)

Despite the modern trends coming from Western cultures, the traditional way of living is one of the most important values of the population in all segments of life. Consanguinity is not common among members of the two main religious groups. However, a rare occurrence of marriages among distant relatives has been observed in isolated and small communities located away from the urban areas.

## 2 POPULATION HISTORY

As a crossroads between the Eastern and the Western cultures, migration of the population in this area represented a continuous process through the centuries, supported by frequent military conflicts and wars. Population diversity in the Balkan Peninsula dates back to 8th to 9th century BC (Folk, 1973), a period when the Balkans was inhabited by the Peony tribe; followed by Hellenistic, Illyrian, and Slavic influence consecutively. Some of the more recent genetic studies based on the analysis of HLA haplotypes, Y‐DNA, and mt‐DNA (Arnaiz‐Villena et al., 2001 ; Jakovski, 2011; Petlichkovski et al., 2004) indicate that Macedonians originate from “an older Mediterranean lineage.” Studies concerning the genetic markers of the Y chromosome in the Balkan region show that the majority of the Balkan population share the equal genetic pool as the Europeans (Kovacevic et al., 2014; Noveski, Trivodalieva, Efremov, & Plaseska‐Karanfilska, 2009; Mirabal et al., 2010).

The Albanian population is the second most prevalent living in the Republic of Macedonia and it is culturally, demographically, and linguistically distinct from other populations in the surrounding countries. They are believed to descend from the so‐called Indo‐European group of nations. Studies based on the analysis of blood group distribution (ABO, MN, and Rh) suggest a diversity in relation to the neighboring nations (Susanne, Bajrami, Kume, & Mikerezi, 1996), while more recent studies based on DNA technologies indicate certain genetic similarity of Albanians with other Europeans (Belledi et al., 2000). However, when using the principal component analysis (PCA), they position as a more distant cluster than other European nations. (Coklo et al., 2016).

## 3 THE HEALTHCARE SYSTEM IN GENERAL

Based on the Law on Health Protection, Macedonia has a social healthcare system, where all citizens have the right to health insurance, which provides them access to both basic and advanced healthcare services, regardless of their income and contribution to the healthcare fund (Parliament, Law on Healthcare, Official Gazette 39, 2014). At present, the contribution to the healthcare fund is up to 7% of the monthly income of each employed citizen, while the services are used by all citizens depending on their need, including the unemployed who have equal access to the services in all health systems. The healthcare is based on the unity of preventive, diagnostic‐therapeutic and rehabilitation measures based on the principles of availability, efficiency, continuity, fairness, and comprehensiveness. The funds for healthcare activities in the network are provided from the budget of the Republic of Macedonia. Efforts are being made to establish private, currently voluntary pillars of additional healthcare insurance in the recent years.

Most of the services are provided in public health institutions, structured in a network of three levels—primary, secondary, and tertiary. Governmental priorities are aimed at ensuring high‐quality and safe healthcare treatment and specifically, in reducing infant mortality, infectious diseases, malignant diseases, etc.

## 4 GENETIC SERVICES AND POSSIBILITIES FOR GENETIC TESTING

Legislation relating the genetic diseases and genetic testing is provided in the Law on the Protection of Patients’ Rights (Parliament, Law on Healthcare, Official Gazette 82, 2008). According to this law, the tests focused on genetic diseases are carried out only to achieve a certain healthcare aim. Scientific research in the field of genetic testing is primarily conducted to fulfill a certain healthcare goal. Actions on human genome for other than preventive, diagnostic, and therapeutic purposes without proper genetic counseling are forbidden.

## 5 POSSIBILITIES OF GENETIC TESTING IN THE PAST AND TODAY

Early stages of genetic testing in Macedonia date back to the 1970s with the establishment of two independent cytogenetic laboratories—one at the University Clinic for Children's Diseases (mainly dedicated to postnatal karyotyping) and consecutively, at the University Clinic for Gynaecology and Obstetrics (for prenatal karyotyping). The first karyotyping was performed in 1972 at the University Clinic for Children's Diseases, while the prenatal analyses were introduced at the University Clinic for Gynaecology and Obstetrics (amniocentesis since 1976, chorion biopsy since 1990). Along with the laboratory tests, a genetic counseling service is organized at both clinics (Table 2).

**Table 2:** Time frame of introduction of cytogenetics analyses in Macedonia

Karyotyping	Karyotyping Prenatal	Karyotyping of malignant diseases	FISH microdeletions	FISH for oncogenetics
1972	1979	1983	2001	2012

The molecular diagnostics in Macedonia primarily started on animal samples (polymorphism of hemoglobins in sheep, 1967) and later has been translated to the human genome. The first laboratory for genetic analysis of hemoglobinopathies was established within the University Clinic for Children's Diseases in 1973, which soon grew into a reference center for hemoglobinopathies in Yugoslavia and the Balkans. Based on population studies carried out on the territory of former Yugoslavia, many new types of hemoglobin (some of them unique) have been discovered and published (Efremov, 2011). The Research Centre for New Technologies with a main focus on introducing new molecular techniques was established in 1986 within the Macedonian Academy of Sciences and Arts. It soon became the Research Centre for Genetic Engineering and Biotechnology, a worldwide well‐known institution. Besides being a core of scientific research in the field of genetics, this center was also a core of introducing new genetic technologies and education. Starting in the new Millennium, several new genetic laboratories have emerged:

- Institute of Immunobiology and Human Genetics—involved in testing of immunogenetic polymorphisms, population studies, testing of genes associated with thrombophilia, familial Mediterranean fever, hereditary hemochromatosis, cystic fibrosis, and other frequent genetic disorders like lactose intolerance or celiac disease.
- Faculty of Pharmacy, Center for Biomolecular Pharmaceutical Analyses—exploring genomic variants responsible for efficacy/toxicity of therapeutic agents and detection of genetic changes in leukemia, colon, thyroid, breast cancer, etc.
- Molecular laboratory at the Institute of Biology, performing HPV genotyping
- Genetic laboratory for forensic medicine—determination of paternity, forensic analysis of human remains, population studies on mt‐ and Y chromosome DNA.
- Molecular laboratory at the Institute of Pathology, analyzing oncologic samples.
- Certain genetic services are offered by a number of private laboratories, partnering with many European renowned genetic laboratories (Synlab, Genica, Acibadem), primarily intended to confirm less common or rare genetic disorders. In addition, whole genome and exome sequencing are being introduced and are available at present. However, due to the limited healthcare budget, they are used mainly by patients who can independently finance them. Table 3 summarizes the currently available genetic tests in Macedonia.
  **Table 3:** Genetic testing available in Macedonia

Karyotyping (pre‐ and postnatal)	Brest cancer testing
Karyotyping for oncology (blood, bone marrow, tissue)	Lung cancer testing RT PCR for viral load determination
FISH for microdeletion/duplication	Colorectal cancer testing
QF ‐PCR for prenatal testing	Familial adenomatous polyposis
FISH for oncology	Thyroid cancer testing
CGH	Urogenital cancer testing
Paternity testing	HLA gene polymorphism
Male infertility/Y chromosome deletions	KIR gene polymorphism
Fragile X syndrome	Thrombophylic genes
Nonsyndromic deafness	Schizophrenia polymorphism
Monogenic diseases:	
‐ cystic fibrosis ‐ thalassemia ‐ hemophilia ‐A, ‐B ‐ hemochromatosis ‐ Fanconi anemia ‐ polycythemia ‐ Duchene/Becker muscular dystrophy ‐ spinal muscular atrophy ‐ Myotonic dystrophy ‐ Fridreich's ataxia ‐ Huntington chorea ‐ tuberous sclerosis ‐ adrenoleukodystrophy ‐ alpha 1 antitrypsin deficiency	‐ cystinuria ‐ phenylketonuria ‐ galactosemia ‐ Lech Nichan syndrome ‐ Vit D‐resistant rickets ‐ neurofibromatosis 1/2 ‐ congenital adrenal hyperplasia ‐ androgen receptor gene mutations ‐ Nijmegen breakage syndrome ‐ Balkan endemic nephropathy ‐ Gilbert syndrome ‐ Wilson disease ‐ Darier disease ‐ Gaucher's disease ‐ Familial Mediterranean fever ‐ achondroplasia

## 6 ORGANIZATION OF GENETIC SERVICES IN MACEDONIA TODAY

Genetic services in Macedonia are an integral part of the overall healthcare system. Most of the genetic services in the public healthcare are covered by the Health Insurance Fund and the users participate with 10% of the total cost of healthcare service. Implementation of new genetic tests in the public health institutions is constantly discussed among the Health Insurance Fund, professionals, patients, and their NGO‐s. However, the Fund still does not cover the costs of genome or exome sequencing which is available only by self‐financing so far. Since 2009, a rare diseases program exists within the Ministry of Health, funding for molecular detection of rare diseases, primarily aimed for conditions that have a possibility for orphan drug therapy or are suitable for prenatal diagnostics.

## 7 GENETIC SERVICES IN UNIVERSITY CLINICAL HOSPITALS

Generally, patients with genetic disorders are referred in two clinics in Macedonia:

- University Clinic for Gynaecology and Obstetrics—Risk Pregnancy Department, covering procedures for screening and genetic testing of fetuses. A genetic laboratory works within the Clinic, primarily focused on prenatal karyotyping.
- University Clinic for Children's Diseases—Department of Endocrinology and Genetics, where all children with syndromic or genetic diseases are transferred for diagnosis and consultation. Dysmorphic evaluation of syndromic disorders is performed using LDDB, Possum, and since recently the Face2Gene program.

The genetic laboratory within the later clinic covers cytogenetic testing for conventional karyotyping and FISH testing for microdeletions and subtelomeric deletions, as well as molecular analysis for diagnosis of congenital adrenal hyperplasia, SRY gene analysis, etc. Oncogenetic examinations are also performed, primarily karyotyping for blood and bone marrow disorders, as well as FISH preparations that cover MYC proto‐oncogene, and fusion probes whenever needed at the Oncology Departments for children and adults. Neonatal screening operates within the laboratory; since 2006 all newborns in the country are screened for hypothyroidism as part of the National Program for Mothers and Children's Care. As for the remaining metabolic diseases (disruption of amino acids, organic acid, and fatty acid oxidation disorders), a selective screening for forty conditions is available, with a plan to be included into a program for all neonates in near future.

A genetic counseling service for patients and families with genetic disorders is organized within both institutions. Genetic counseling follows world and European norms and recommendations for counseling high‐risk patients and families.

## 8 EDUCATION IN GENETICS—FORMAL AND INFORMAL

Knowledge accumulation in the field of human genetics has penetrated into every cell of the basic and clinical medicine, and has imposed the need for specific in‐depth education of medical students and related professionals in this field.

Informal education in genetics of professionals involved in a range of related disciplines started like elsewhere in the world—with targeted training of the existing staff members—pediatricians, gynecologists, biologists, pharmacists, etc. Initially, undergraduate education in genetics was included in the study of biological sciences at the Faculty of Biology in Skopje. Specialists in various areas—pediatrics, gynecology, and oncology—had an opportunity to complete subspecialization in genetics.

Comparing the curricula of universities around the world, an initiative to organize formal education in this field of genetics was started. The Cathedra of Human Genetics introduced theoretical and practical teaching for medical students since 2005, although it was officially constituted in 2011, according to the Rulebook of work at the Medical Faculty, accepted at session of the Teaching‐Scientific Council (Medical‐Faculty, 2011).

The course Essentials in Human Genetics has been listed as a mandatory subject of the studies in medicine, and is generally divided into two sections—basic, covering all aspects of DNA structure and processes of transmitting genetic information, and a clinical section integrating the basic laws of inheritance, monogenetic and polygenetic diseases, as well as the methods for their detection. In addition to the compulsory program, two elective courses in this field were developed to supplement the knowledge in this area—Evolution Genetics and Congenital and Hereditary Diseases, where interested students gain additional knowledge in some topics of this field.

One of the goals of the Cathedra was to initiate a formal specialization training in the field of human genetics for physicians. A new speciality training program in clinical genetics for physicians under the guide of the European Society for Human Genetics was launched only in 2015 to complement the already existing specialization in laboratory genetics available for biologists and similar medical support staff members. At present, there are about 20 specialists in laboratory genetics, primarily biologists working in laboratories. In the same time, education of the first generation of medical doctors residents in clinical genetics is under way.

The Macedonian Society of Human Genetics has been recently established, bringing together all professionals working in different fields of clinical or laboratory genetics. The Society aims to adopt common strategies addressing the genetic conditions, and expanding the opportunities for genetic analysis in the country to follow the European and international trends.

## 9 SCIENTIFIC RESEARCH ACTIVITY

The first scientific research in the field of genetics in Macedonia dates back to more than 30 years ago, with the study on the prevalence of blood groups in the skeletal remains from the archeological site Stobi (Stojanovski, 1982). First genetic diseases in the region detected as thalassemia have been proven in the calvarias found in the same archeological site (Wesolowsky, 1983), on remains dating from around 2nd century B.C. according to widened and distorted diploic space of the skulls in some skeletons.

Contemporary scientific research in genetics continued with the establishment of institutions and laboratories, and encompassed dysmorphology, cytogenetics, oncogenetics, molecular cytogenetics, etc. (Kočova & Sandberg, 1985; Sukarova‐Angelovska, 2002; Sukarova‐Angelovska, Kocova, Ilieva, Angelova, & Sredovska, 2005; Sukarova‐Angelovska, 2002). In the field of immunogenetics, a series of studies have been published in well‐known journals (Petlichkovski et al., 2004, 2011; Petlickovski et al., 2005;Spiroski et al., 2013). The research studies published by the Research Center for Genetic Engineering and Biotechnology regarding the molecular diagnostics of many monogenetic diseases and chromosomal disorders are numerous, some describing unique genetic variants (Efremov, Dimovski, & Huisman, 1994; Efremov et al., 1988, 1996; Madjunkova, Kocheva, & Plaseska‐Karanfilska, 2014; Dimovski AJ, A large beta‐thalassemia deletion in a family of Indonesian‐Malay descent., 1996; Plaseska et al., 1990).

The number of ongoing and completed PhD theses at the Sv. Kiril i Metodij University based on genetic research at the Cathedra of Human Genetics is rapidly growing based on genetic research is rapidly growing. Participation in a range of domestic and international scientific research projects in the field of genetics gives an insight into significant cooperation of geneticists in the country and abroad. Some well‐known international organizations, such as ICGEB (seeded in Trieste, Italy), Erasmus and Tempus (European Union program for support of education and training), Italian National Research Centre “Adriano Buzzati,” and Institute for Human Genetics in Lueven (Belgium) lead some of these projects.

In addition, researchers from Macedonia have presented their work at a range of international and domestic congresses and expert meetings in the field of genetics. More than 100 papers have been published in international and domestic journals covering the field.

## 10 CONCLUSION

Modern genetics is a science with rapid development. In recent years, a comprehensive new knowledge about the pathogenic mechanisms of many genetic diseases and potential new treatments has come to light. Despite the limited healthcare and scientific resources, Macedonia manages to follow the new trends in genetic diagnostics and to modernize the legislation related to the genetic examinations and research. Considering that medical genetics penetrates all fields of medicine and can modify the approach toward certain diseases from its root, the healthcare professionals along with affected patients, families, and authorities in the country make efforts for a comprehensive coverage of the genetic diseases and conditions.
